# Time-dependent patterning of the mesoderm and endoderm by Nodal signals in zebrafish

**DOI:** 10.1186/1471-213X-7-22

**Published:** 2007-03-28

**Authors:** Engda G Hagos, Scott T Dougan

**Affiliations:** 1 Department of Cellular Biology, The University of Georgia, Athens, GA, USA

## Abstract

**Background:**

The vertebrate body plan is generated during gastrulation with the formation of the three germ layers. Members of the Nodal-related subclass of the TGF-β superfamily induce and pattern the mesoderm and endoderm in all vertebrates. In zebrafish, two *nodal-related *genes, called *squint *and *cyclops*, are required in a dosage-dependent manner for the formation of all derivatives of the mesoderm and endoderm. These genes are expressed dynamically during the blastula stages and may have different roles at different times. This question has been difficult to address because conditions that alter the timing of *nodal-related *gene expression also change Nodal levels. We utilized a pharmacological approach to conditionally inactivate the ALK 4, 5 and 7 receptors during the blastula stages without disturbing earlier signaling activity. This permitted us to directly examine when Nodal signals specify cell types independently of dosage effects.

**Results:**

We show that two drugs, SB-431542 and SB-505124, completely block the response to Nodal signals when added to embryos after the mid-blastula transition. By blocking Nodal receptor activity at later stages, we demonstrate that Nodal signaling is required from the mid-to-late blastula period to specify sequentially, the somites, notochord, blood, Kupffer's vesicle, hatching gland, heart, and endoderm. Blocking Nodal signaling at late times prevents specification of cell types derived from the embryo margin, but not those from more animal regions. This suggests a linkage between cell fate and length of exposure to Nodal signals. Confirming this, cells exposed to a uniform Nodal dose adopt progressively more marginal fates with increasing lengths of exposure. Finally, cell fate specification is delayed in *squint *mutants and accelerated when Nodal levels are elevated.

**Conclusion:**

We conclude that (1) Nodal signals are most active during the mid-to-late blastula stages, when *nodal-related *gene expression and the movement of responding cells are at their most dynamic; (2) Nodal signals specify cell fates along the animal-vegetal axis in a time-dependent manner; (3) cells respond to the total cumulative dose of Nodal signals to which they are exposed, as a function of distance from the source and duration of exposure.

## Background

During vertebrate development, cells become irreversibly committed to particular fates after a series of inductive events. The first step of this process is completed during gastrulation, when cells are allocated to the three germ layers. Fate maps of vertebrate embryos show considerable organization before gastrulation, since different mesodermal and endodermal structures are derived from distinct positions along the major body axes [1-3]. In zebrafish late blastula stage embryos, for example, endoderm progenitors are restricted to the four rows of cells closest to the yolk, known as the margin, while mesoderm precursors extend further towards the animal pole [4,5]. The germ layers are also patterned along the dorsoventral axis, such that the notochord is derived from dorsal mesoderm, the heart comes from lateral mesoderm and blood comes from ventral mesoderm [6,7]. TGF-β signals of the Nodal-related subclass are required to induce and pattern the germ layers in vertebrates [8]. Nodal signaling is mediated by a receptor complex containing the TGF-β Type I receptor, ALK4, the Type II receptor, ActR-IIB, and the Cripto/One- Eyed-pinhead (Oep) co-receptor [9,10]. The Nodal receptors can also be activated by other TGF-β ligands, including Activin and Vg1 [9,11]. For this reason, the Nodalrelated proteins, Activin and Vg1 are collectively termed Activin-like signals.

The requirement for Nodal-related proteins to induce mesoderm and endoderm is conserved throughout the vertebrate lineage [8]. There are three *nodal-related *genes in zebrafish, but only two, *squint *(*sqt/ndr1*) and *cyclops *(*cyc/ndr2*), have overlapping roles in mesendoderm formation [12]. The third *nodal-related *gene, *southpaw *(*spaw/ndr3*), is only expressed after gastrulation and is involved in establishing the left-right body axis [13]. In *cyc *single mutants, defects in mesendoderm are first detected at mid-gastrulation and the embryos lack floorplate and ventral diencephalon at later stages [14-16]. *sqt *single mutants have severe deficits in dorsal mesodermal derivatives at early stages, but the embryos recover and many survive to adulthood [17,18]. This recovery depends on *cyc *function, since *sqt; cyc *double mutants lack all derivatives of the mesoderm and endoderm in the head and trunk, including the skeletal muscle, heart, pronephros, blood and gut [19].

Both gain-and loss-of-function studies indicate that Activin-like signals act in a concentration-dependent manner to specify cell fates [20-23]. In explants, high doses induce marginal cell types, such as prechordal plate and endoderm, whereas lower doses induce notochord and muscle [24]. Conversely, endoderm and prechordal plate are more sensitive to reductions in Nodal levels than are notochord and muscle [17,23]. Zebrafish Sqt behaves like a morphogen, acting directly on cells at a distance to specify fates in a concentration dependent manner [25,26]. These results and other data have led to the suggestion that cells adopt fates depending on their position within a gradient of Nodal-related protein [27].

A spatial gradient model of Nodal signaling, however, does not account for two key observations. For example, in the animal region of the mesoderm territory in pregastrula stage embryos, somite precursors are intermingled with neurectoderm progenitors, which are specified in the absence of Nodal function [5,17]. Near the margin, by contrast, somite precursors are intermingled with endoderm precursors, which require high levels of Nodal [4,17]. This raises the question of how adjacent cells could be exposed to different Nodal doses. Secondly, Cyc can fully compensate for loss of the Sqt morphogen despite the fact that it only acts over a short range [17,25,28]. This indicates that the long-range action of Nodal signals is not necessary for correct induction and patterning of the mesoderm and endoderm.

Experiments suggest that the role of Nodal signaling is quite dynamic, but it has been difficult to determine what are the functions of Nodal signals at different times. The expression pattern of *nodal-related *genes changes rapidly during the blastula stages in frogs, fish and mice [21,24,29,30]. Efforts to determine when Nodal signals specify distinct mesodermal and endodermal cell types have been hampered by the fact that conditions which alter the timing of Nodal signaling also change the levels of *nodal related *gene expression. For example, levels of Nodal decrease in zygotic *oep *mutants as maternally supplied Oep mRNA and protein decay and eventually disappear [31,32]. Similarly, *cyc *expression is both reduced and delayed in *sqt *mutants [17]. Thus, it has not been possible to determine whether the fate changes observed in these mutants are due to altered timing of Nodal signaling or to the reduction in Nodal activity.

Experiments in frogs and fish have suggested two mechanisms by which Nodal signals may act to specify different tissues at different times. When *Xenopus *animal cap cells are exposed to Activin-soaked beads for different lengths of time, the responding cells exhibit a stepwise progression of cell fate specification as a concentration gradient of Activin is established within the explant [33]. These results suggested that cells constantly monitor ligand levels and "ratchet-up" their response when the concentration exceeds certain threshold levels. In this view, cell fates are determined by the absolute number of receptors occupied by the ligand rather than by how long cells are exposed to the ligand [34,35]. By contrast, experiments in zebrafish with a conditional allele of *cyc *determined that cells need to be exposed to Nodal signals during a two-hour window in order to become floorplate [36]. This raised the possibility that cells respond differently to Nodal signals depending on when they are exposed. In this view, cells have intrinsically defined periods during which they are able to adopt particular fates if exposed to the proper Nodal dose.

We have utilized a pharmacological approach to determine when Nodal signals specify the different mesodermal and endodermal cell types in the zebrafish. For the first time, we have been able to block the activity of Nodal receptors during discrete blastula stages by treatment with the small molecules SB-431542 or SB-505124 and without disrupting signaling at earlier stages or altering endogenous Nodal levels [37,38]. We find that Nodal signals specify most mesodermal and endodermal cell types between the mid-blastula (3 h) and late blastula (5 h) stages. By examining embryos with increased or decreased levels of Sqt and Cyc signals, we show that the Nodal dose controls the timing of cell fate specification. This rules out the idea that cells adopt different mesoderm and endoderm fates depending on when they are exposed to Nodal signals. We also show that embryonic cells respond to a uniform, high dose by adopting progressively more marginal fates with longer exposures to Nodal signals. This time-dependent transformation of cell fates is inconsistent with some aspects of the ratchet model. We conclude that cells respond to the total cumulative dose of Nodal signals to which they are exposed, as a function of distance from the source and duration of exposure.

## Results

### Drug treatment at MBT prevents the response to zygotic Nodal signals in embryos

**Figure 1:**
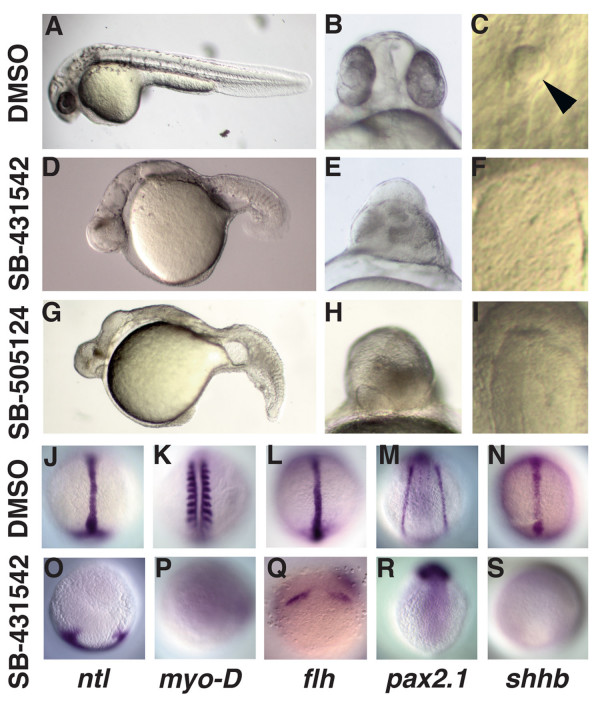
**Treatment with 800 μM SB-431542 or 50 μM SB-505124 at MBT prevents formation of mesoderm and endoderm**. (A-F) Images of live embryos at 24hpf treated at 2.75 h with DMSO (A-C), SB-431542 (D-F; J-S), or SB-505124 (G-I). Embryos treated with SB-431542 (D-F) or SB-505124 (G-I) lack derivatives of the mesoderm and endoderm in the head and trunk, display severe cyclopia and lack Kupffer's vesicle. (J-P) Images of embryos treated with DMSO (J-N) or SB-431542 at MBT (O-S) and processed to reveal expression of markers for derivatives of dorsal mesoderm (*ntl*: J, O; *flh*: L, Q), paraxial mesoderm (*myoD*: K, P), intermediate mesoderm (*pax2.1*: M, R), and ventral neurectoderm (*shhb*: N, S). Dorsal views of embryos fixed at 10 h (J, L, N, P, Q, S)or 14 h (K, M, P, R). Arrowhead in (C) is the Kupffer's vesicle.

To determine when Sqt and Cyc signals induce and pattern the germ layers, we developed a drug-based strategy that permits us to block endogenous Nodal signals at different stages after the mid-blastula transition (MBT). SB-431542 binds competitively to the ATP binding sites of the ALK 4, 5 and 7 receptors, preventing their kinase activity [39]. This drug has been used previously on zebrafish embryos during the cleavage stages, but did not completely block Nodal signals when added after MBT [40,41]. Therefore, we developed a protocol to use SB-431542 to block zygotic Nodal signals in whole embryos between MBT (2.75 h) and the onset of gastrulation (6 h) (see Methods). Control embryos had a normal morphology at 24 h, indicating that our manipulations did not affect early embryogenesis (Fig. 1A–C). By contrast, embryos treated with 800 μM SB-431542 display severe cyclopia and lack all derivatives mesoderm and endoderm in the head and trunk, including the somites, notochord, blood, heart and Kupffer's vesicle (Fig. 1D–F). These defects strongly resemble those previously described for *sqt; cyc *double mutants [19]. Like *sqt; cyc *double mutants, SB-431542 treated embryos lack axial expression of the pan-mesendodermal marker *no-tail *(*ntl*) and the notochord marker *floating head *(*flh*) (Fig. 1J, L, O, Q). Interestingly, *flh *expression in the neurectoderm is greatly expanded in drug treated embryos, suggesting an expanded epiphysis (Fig. 1Q) [42]. Drug treated embryos also lack *MyoD *expression at 14 h (Fig. 1K, P). Since tail somites do not form until later stages, this indicates that trunk somites are missing [43]. The prechordal plate and pronephros are also missing in these embryos, as indicated by the lack of *goosecoid *(*gsc*) and *pax2.1 *expression, respectively (Fig. 1R, M; Fig. 3C, D). Drug treated embryos also lack expression of *sonic hedgehog b *(*shhb*), indicating the absence of floorplate (Fig. 1N, S). Because high concentrations of the drug were necessary to produce these defects, we next asked if we could achieve similar results with SB-505124, a more potent and bioactive inhibitor of the ALK 4/5/7 receptors than SB-431542 [38]. 30–50 μM of SB-505124 is sufficient to phenocopy *sqt; cyc *mutants when added at MBT (Fig. 1G–I). The ability of both drugs to phenocopy *sqt; cyc *mutants when added to 2.75 h embryos indicates that they reduce ALK 4/5/7 receptor activity to levels as low as that in zygotic mutants null for *nodal-related *gene function. Subsequent experiments were performed with SB-431542 and confirmed with SB-505124 as indicated.

**Figure 2:**
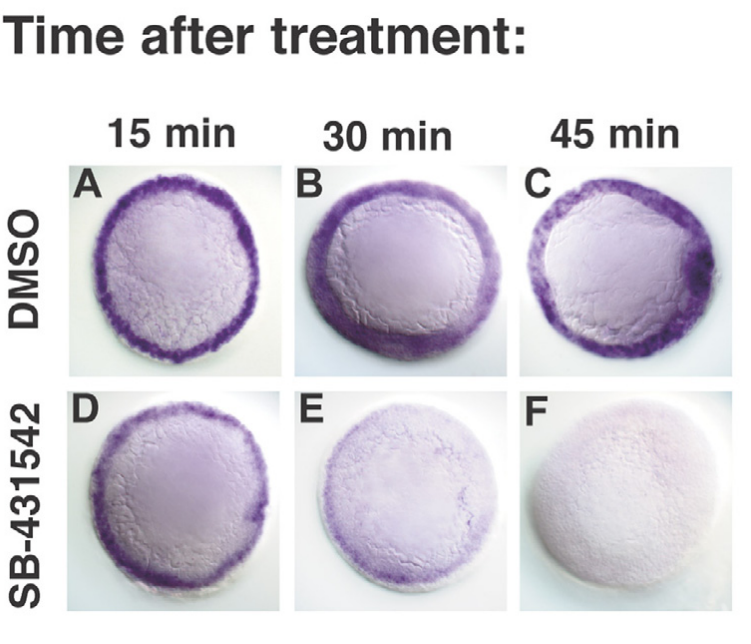
**SB-431542 rapidly blocks transcription of Nodal target genes**. *lefty1 *expression in embryos treated with DMSO (A-C) or SB-431542 (D-F) at 4.3 h (dome stage), and fixed after 15 minutes (A, D), 30 minutes (B, E) or 45 minutes (D, F).

To determine how quickly we could observe the effects of the drug, we examined the expression of the Nodal target gene *lefty1 *in a time course of embryos treated with SB-431542 at dome stage (4.3hpf) [44]. We found that transcription of Nodal target genes is normal 15 minutes after treatment (Fig. 2A, D; N = 11), but is severely reduced after 30 minutes (Fig. 2B, E; N = 24). No transcripts are detected 45 minutes after treatment (Fig. 2C, F; N = 21). Therefore, transcription of Nodal-dependent genes is rapidly blocked after drug treatment and the decrease in mRNA levels is apparent within 15–30 minutes.

**Figure 3:**
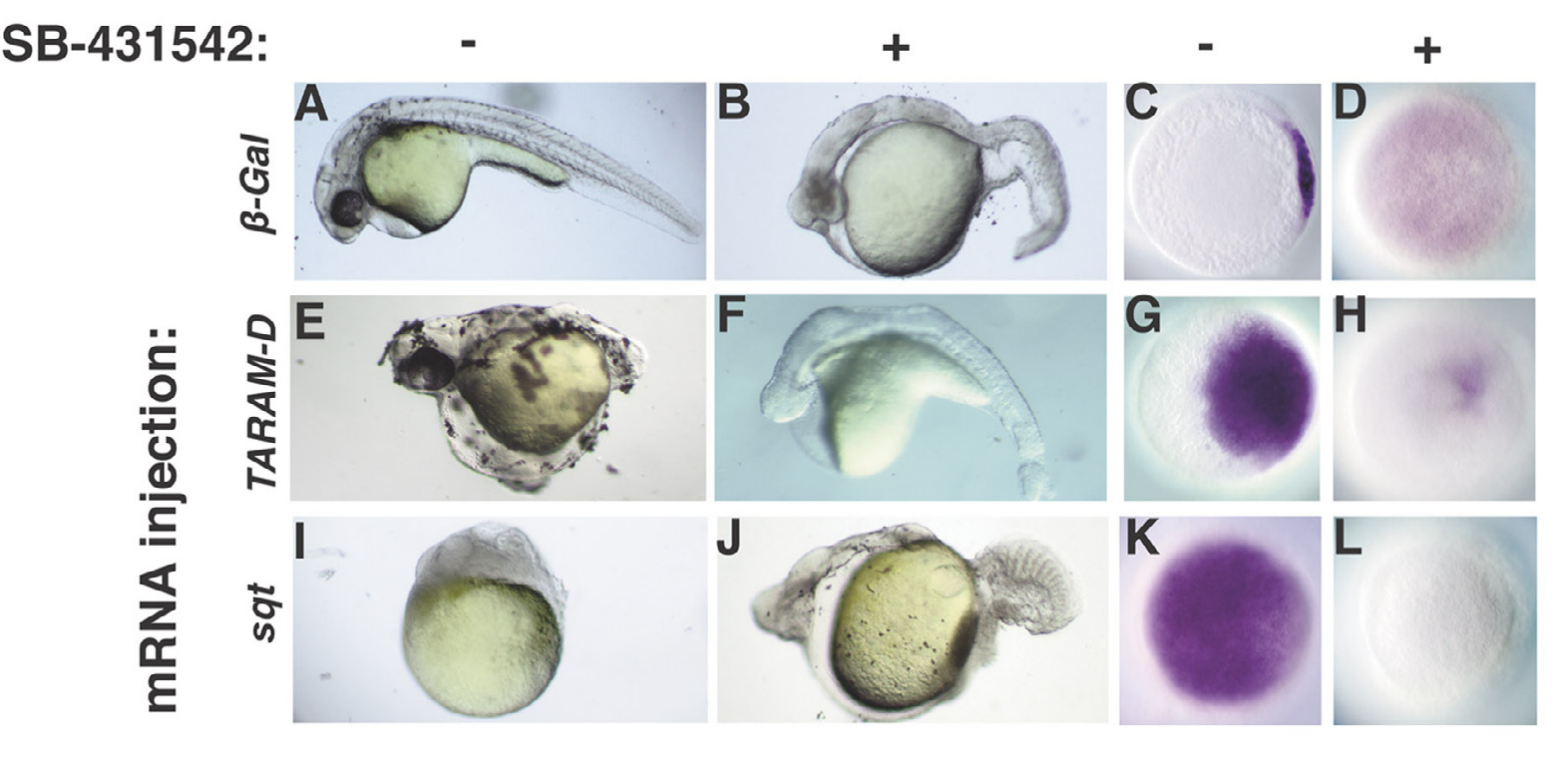
**Treatment at MBT blocks the response to receptors activated during the cleavage stages**. Embryos injected at the 1–4 cell stage with 10pg *β-galactosidase *(A-D), *TARAM-D* (E-H), or *sqt *(I-L) mRNA, and treated at 2.75 h with DMSO (A, E, I, C, G, K) or SB-431542 (B, F, J, D, H, L). (E, G) *TARAM-D *induces ectopic body axes and *gsc *expression. (F, H) The effects of *TARAM-D *are suppressed by treatment with SB-431542. (I, K) *sqt *overexpression arrests epiboly and induces ubiquitous expression of *gsc*. (J, L) The response to ubiquitous Sqt is blocked by treatment with SB-431542. Images of live embryos at 30 h, anterior to the left (A, B, E, F, I, J). Animal pole views of fixed embryos at 5 h, dorsal to the right (C, D, G, H, K, L).

We next asked if SB-431542 could prevent the response to a mutated and constitutively-activated receptor that is active even in the absence of ligand, such as *TARAM-D *[39]. *TARAM-D *acts in a cell-autonomous manner to induce expression of Nodal target genes, resulting in dorsalized embryos and expanded *gsc *expression (Fig. 3E, G; N = 30) [28,45]. In most cases, SB-431542 completely suppresses the response to *TARAM-D*, consistent with its proposed mode of action (data not shown). In the course of our experiment, however, occasional embryos received higher doses of the activated receptor and displayed a milder phenotype than their siblings. These embryos have cyclopia and reduced or absent mesodermal tissues, including trunk somites and notochord (Fig. 3F). *gsc *expression is dramatically reduced in these embryos (Fig. 3G, H; N = 20). Thus, high levels of activated receptor can rescue the defects caused by the drug. This demonstrates the specificity of the drug, since the activated Nodal receptor would not rescue defects caused by blocking receptors for other signaling pathways. SB-431542 also blocks the response to ubiquitously expressed Sqt (Fig. 3I–L). Thus, the drug is able to effectively penetrate and act within the entire embryo. In these experiments, we injected embryos with *sqt *or *TARAM-D *mRNA at the 1–4 cell stage (1 h) and treated with the drug at 2.75 h. Therefore, SB-431542 can block the response to receptors already present during the cleavage stages. Because the drug is effective at blocking Nodal signaling when applied as late as 2.75 h, this suggests that maternally supplied Activin-like ligands normally act after MBT, if at all, to effect specification of cell fates.

### Nodal signals specify mesodermal tissues at different times within a three-hour period

**Figure 4:**
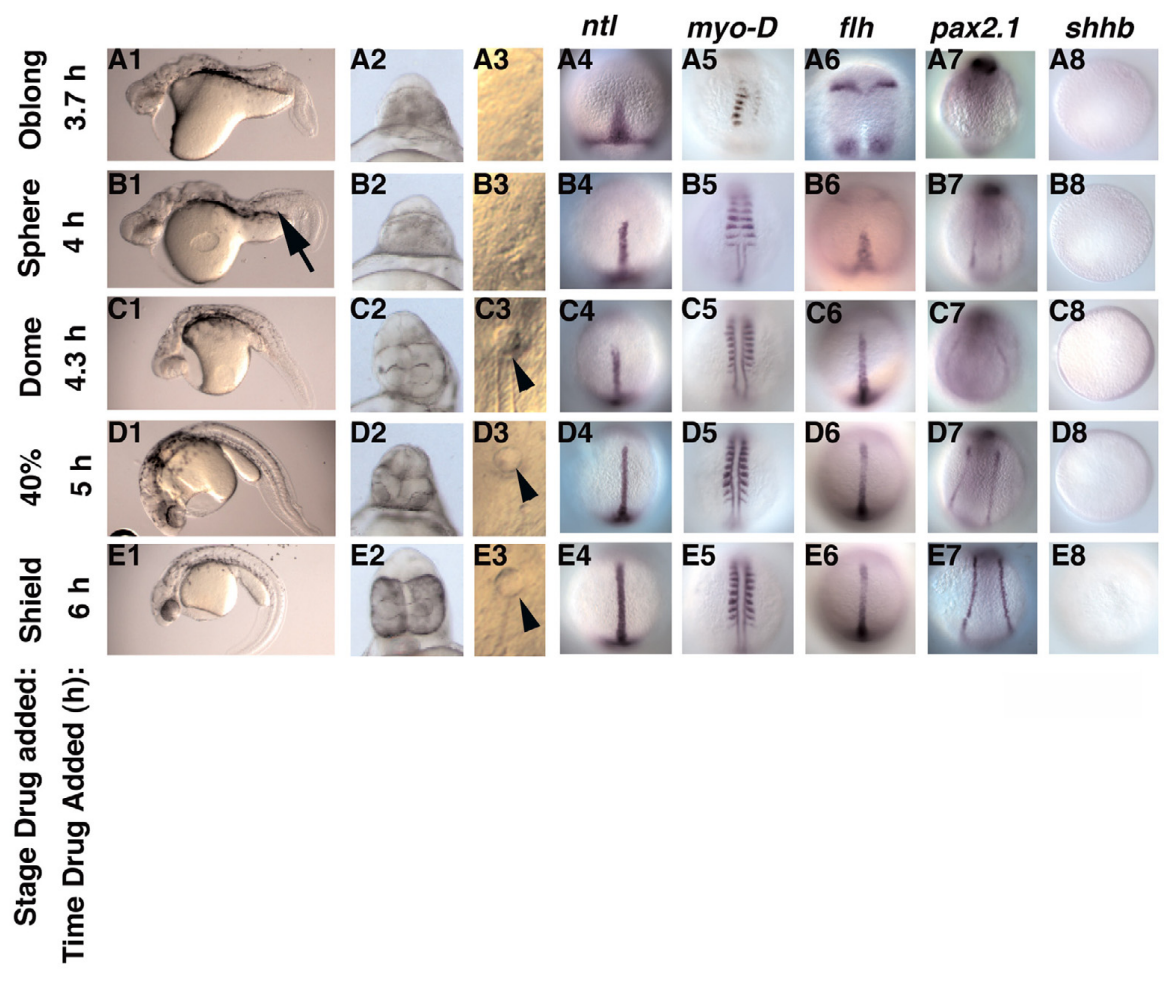
**Nodal signals pattern the mesoderm during a three-hour time window**. (A1-A8) Trunk somites form in embryos treated at 3.7 h (A1, 5), but *flh *is expressed in four ectodermal domains (A6). (B1-B8) Embryos treated at 4 h contain more somites (B5). *flh *is expressed at the midline and small amounts of notochord tissue are observed in live embryos (B1, arrow). *pax2.1 *expression is also observed (B7). At later time points, embryos have progressively more somites and more notochord (C1-E7). *flh *expression extends further up the midline and *pax2.1 *is expressed more strongly. Kupffer's vesicle forms in embryos treated 4.3 h (C3, arrowhead). *shhb *is not expressed in embryos treated before gastrulation (A8-E8). Images of live embryos at 24 h, anterior to the left (A1-E1; A2-E2) or 14hpf (A3-E3); dorsal views of fixed embryos at 10 h (A4-E4; A6-E6; A8-D8) or 14 h (A5-E5; A7-E7). Control embryos are depicted in Fig. 1J-S, which are from the same experiment.

**Figure 5:**
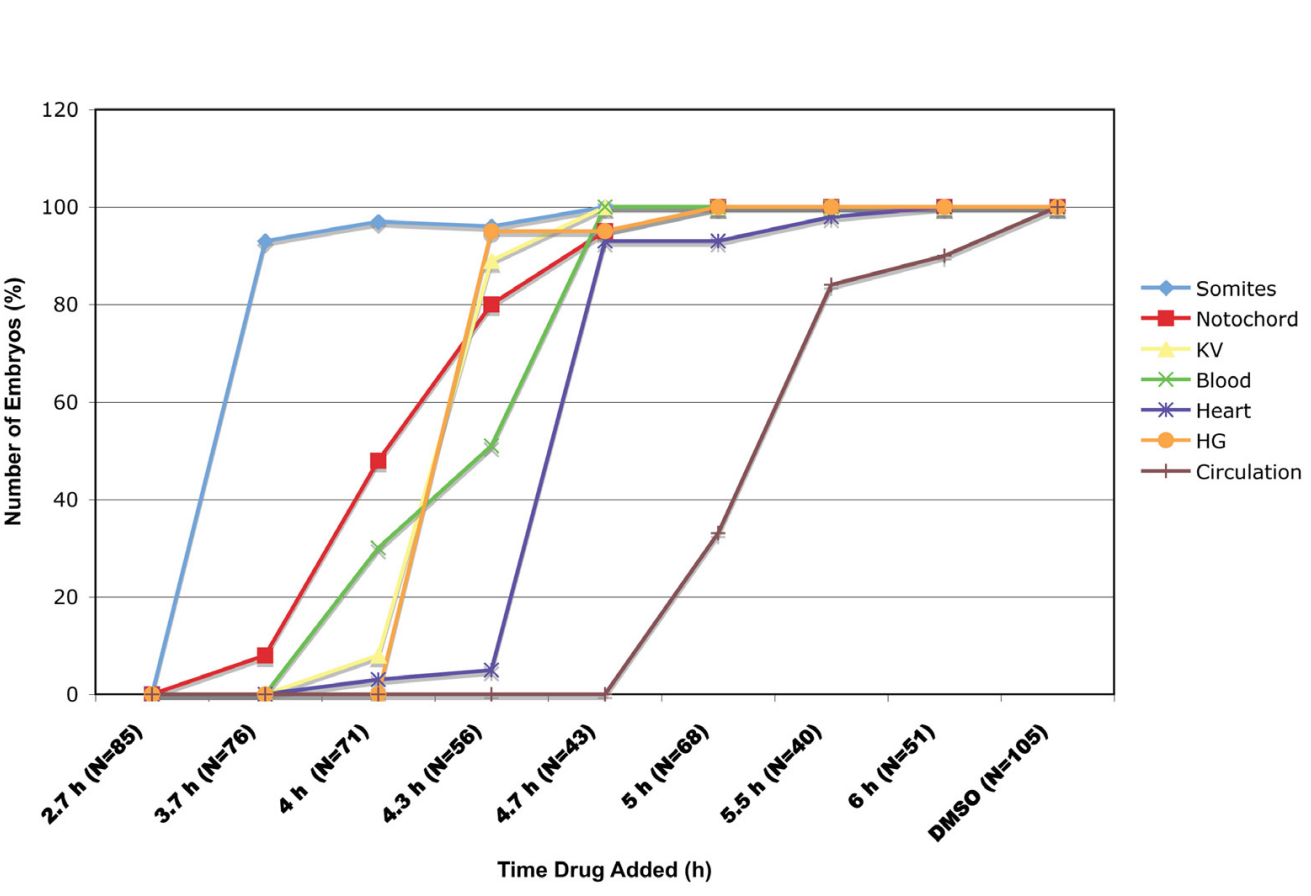
**Time-dependent specification of mesodermal tissues by Nodal signals**. Embryos were treated with SB-431542 at different stages of development between the mid-blastula transition and the onset of gastrulation, and were examined morphologically at 14, 24 and 48 h for visible trunk somites, notochord, blood, Kupffer's vesicle, hatching gland, a beating heart and a functional circulatory system. The steep curves for somites, hatching gland, Kupffer's vesicle, and beating hearts indicates that specification of these tissues occurs rapidly and in a stepwise fashion. Specification of notochord and blood occurs over a longer period. The number of embryos examined at each time point is indicated.

To determine when Nodal signals specify the various mesodermal cell types, we treated embryos with SB-431542 at successively later time points during the blastula stages and scored mesodermal tissues by morphology and marker gene expression. No mesodermal derivatives are present in the head and trunk when embryos are treated with the drug at MBT (Fig. 1). By contrast, embryos treated with SB-431542 at 6 h, when *cyc *expression predominates, produced a phenocopy of *cyc *single mutants (Fig. 4E1-8). These embryos display severe cyclopia, have a ventrally curved body axis and lack the floorplate, as indicated by the absence of *shhb *expression (Fig. 4E1, 2, 8) [14,36]. Thus, SB-431542 treatment in early gastrulation reduces ALK 4/5/7 activity to levels at or below those in *cyc *null mutants. A number of mesodermal cell types are present in embryos treated at this stage, including somites, notochord, heart, blood, pronephros, and hatching gland (Fig. 4E3-7; Fig. 5). This defines a three-hour time window beginning at MBT during which Nodal signals are required to specify mesodermal tissues. Embryos treated after mid-gastrulation contain floorplate as revealed by *shhb *expression (data not shown), confirming earlier temperature shift experiments using a temperature sensitive allele of *cyc *[36].

We next treated embryos with SB-431542 at different times between 2.75 and 6 h post-fertilization. Embryos treated with SB-431542 at 3.7 h contain a small number of trunk somites, but we detected no other mesodermal tissues in the trunk (Fig. 4A1; Fig. 5). *ntl *was expressed in a truncated axial domain and only a small number of disorganized trunk somites are apparent, as indicated by *MyoD *expression (Fig. 4A4, A5). *flh *was expressed in two bilateral domains within the ectoderm, but not at the midline (Fig. 4A6), consistent with the lack of notochord tissue in these embryos (Fig. 4A1, Fig. 5). The expression of the pan-mesodermal marker, *ntl*, but not notochord marker, *flh*, at the midline suggests that these cells are specified to be dorsal mesoderm, but are unable to complete the differentiation program [46,47]. The lack of *pax2*.*1 *expression in the intermediate mesoderm indicates that the pronephros was not specified at this time point (Fig. 4A7). Therefore, only trunk somites were specified following the shortest exposure time to Nodal signals.

Embryos treated with SB-431542 at later time points contain a more diverse array of mesodermal tissues (Fig. 5). Small amounts of notochord are detected in embryos treated at 4 h (Fig. 4B1, arrow; Fig. 5). Red blood cells are also apparent in live embryos examined at 48 h (Fig. 5). *flh *expressing cells populate the midline, but do not induce expression of *MyoD *in adaxial cells within the segmented mesoderm (Fig. 4B4-6). *MyoD *is still expressed in adaxial cells in the presomitic mesoderm (Fig. 4B5). *pax2.1 *expression is also apparent in embryos treated at 4 h (Fig. 4B7). The hatching gland and Kupffer's vesicle are first visible in embryos treated with the drug at 4.3 h (Fig. 4C3, arrowhead; Fig. 5). Although we observed beating hearts in embryos treated at 4.7 h, a functioning circulatory system was only established in embryos treated at 5 h (Fig. 5). Since blood is specified before the heart, we attribute the delay in circulation to the time required to specify the cells comprising the vasculature, although we have not directly examined these cell types. Tissues were specified in the same temporal order in a time course using SB-505124 (data not shown).

The total amount of mesoderm increases as embryos are treated at successively later stages. Embryos treated at 3.7 h have between 5–7 trunk somites (Fig. 4A1, 5). By contrast, embryos treated at the onset of gastrulation contain the normal complement of somites (Fig. 4E1, 5). Thus, new somite tissue is induced throughout the blastula period. Similarly, a truncated notochord forms in embryos treated at 4 h (Fig. 4B1, 6, arrow), but notochord tissue extends more anteriorly when later stage embryos are treated (Fig. 4C1-E1; C6-E6). We were unable to detect a difference in the length of notochord in embryos treated at 5 h and 6 h (Fig. 4D6, E6). Expression of *flh *in the neurectoderm diminishes concomitantly with its expansion along the midline, indicating that signals from the mesoderm inhibit the differentiation of some neural tissues (Fig. 4A6-C6). *pax2.1 *expression is weak when Nodal signaling is blocked at 4 h (Fig. 4B7), but intensifies when Nodal signaling is blocked at later stages (Fig. 4C7-E7). This demonstrates that after 4 h, Nodal signals act to specify the somites, notochord and pronephros, simultaneously. This argues against, but does not completely exclude, a model in which Nodal signals specify different mesoderm and endodermal cell types during distinct time-windows.

### Nodal signals pattern the animal-vegetal axis in a time-dependent manner

**Figure 6:**
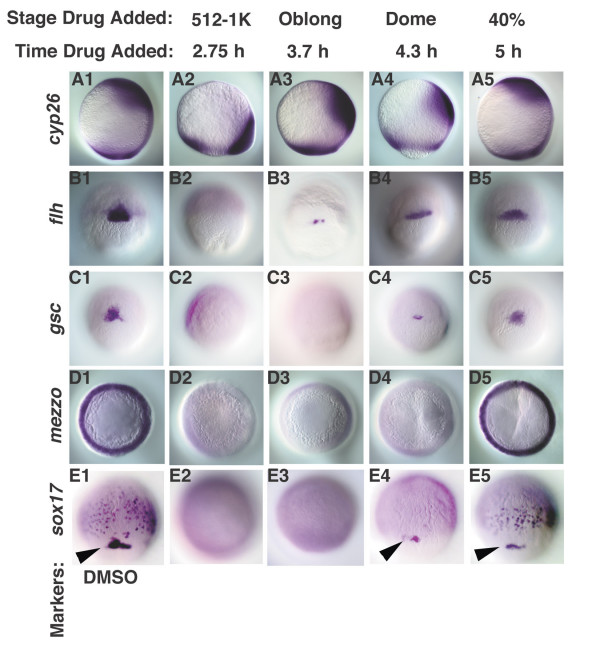
**Nodal signals pattern the dorsal mesoderm and endoderm along the animal-vegetal axis in a time-dependent manner**. Dorsal cell fates were examined in embryos treated with DMSO (A1-E1), or with SB-431542 at various time points. (A2-5) *cyp26 *expression was expressed at the margin in embryos treated at MBT, but is expressed in more animal locations at later time points. (B2-5) *flh *is first detected in embryos treated at 3.7 h. (C2-5) *gsc *is first observed in embryos treated at 4.3 h (dome stage), but is expressed at normal levels in embryos treated after 5 h (40% epiboly). *mezzo *transcripts are observed in embryos treated after 5 h (40% epiboly) (D5), but not at earlier stages (D2-4). *sox17 *is expressed in the dorsal forerunner cells in embryos treated 4.3 h (dome stage) (E4, arrowhead), but is first detected in endoderm progenitors in embryos treated at 5 h (40% epiboly) (E5). Lateral views of embryos at 10 h are depicted in A1-5, dorsal to the right. Dorsal views of embryos at 7 h (60% epiboly) (B1-C5), 5.5 h (germ ring) (D1-5) and 8 h (80% epiboly) (E1-5) are depicted. Arrowheads (E1, 4, 5) indicate *sox17 *in dorsal forerunner cells. All embryos are siblings.

Somite progenitors extend to the most animal region of the mesoderm territory in the pre-gastrula stage embryo, while progenitors of the hatching gland are restricted to the margin [5,6,17]. Thus, our data suggests a general trend in which animal cell types are specified by shorter periods of Nodal signaling than marginal cell types. To test this, we asked when Nodal signals are required to specify the neural plate, notochord, prechordal plate and endoderm, which are marked by expression of *cyp26*, *flh*, *gsc *and *sox17*, respectively (Fig. 6A1-C1, E1). Embryos treated at MBT do not express *flh*, *gsc *or *sox17 *(Fig. 6B2, C2, E2). *cyp26 *is expressed at the margin, consistent with fate mapping studies showing that marginal cells adopt neural cell fates in the absence of Nodal signaling (Fig. 6A2) [12]. This domain shifts toward the animal pole with later treatments, reaching its normal location in embryos treated at 5 h (Fig. 6A2-5). *flh *is first observed at the margin in embryos treated at 3.7 h (Fig. 6B3), but these cells do not differentiate into notochord (Fig. 4A6, N = 20/21). *flh *is expressed at higher levels in embryos treated at later stages and the cells do become notochord (Fig. 6B2-6; Fig. 4B6-E6). *gsc *is not observed in embryos treated at 3.7 h (N = 17), but we detect a small number of *gsc *expressing cells in embryos treated at 4.3 h (Fig. 6C3, C4; N = 9/22). *gsc *is expressed at normal levels in all embryos treated at 5 h (Fig. 6C5; N = 20). This indicates that Nodal signals are required between 4.3 h and 5 h to specify the prechordal plate. Kupffer's vesicles are also specified at this time, as indicated by our analysis of live embryos (Fig. 4C3; Fig. 5) and of *sox17 *expression in the dorsal forerunner cells (Fig. 6E4, arrowhead; N = 17). *sox17 *is expressed in endoderm progenitors in embryos treated at 5 h (Fig. 6E5; N = 15). The paired-box transcription factor *mezzo *acts upstream of *sox17*, and is expressed along the same time course (Fig. 6D1-D5) [48]. On the dorsal side of the embryo, therefore, specification of marginal cell types, but not more animal cell types, is inhibited by late drug treatments.

**Figure 7:**
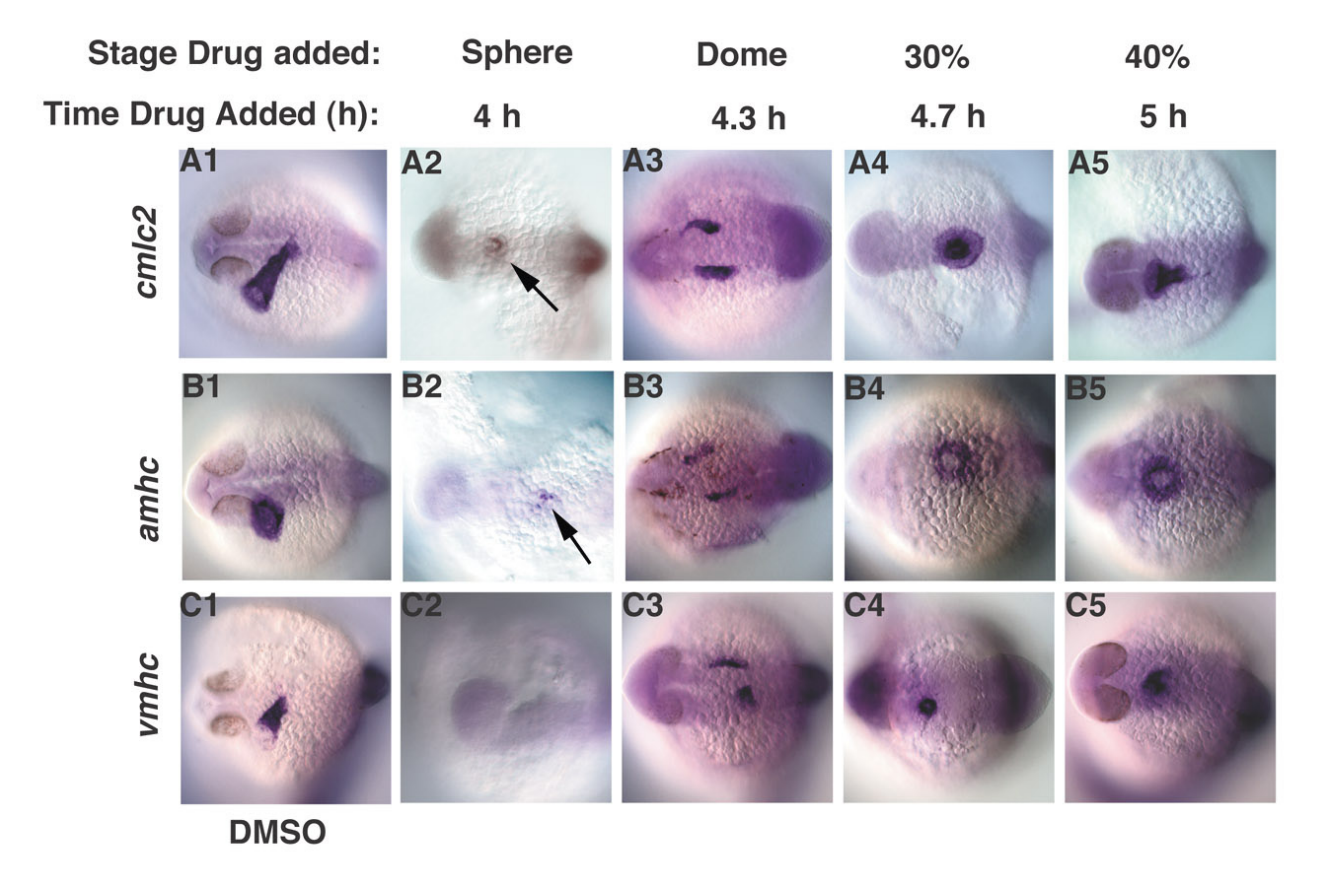
**Nodal signals pattern the ventrolateral mesoderm along the animal-vegetal axis in a time-dependent manner**. Heart myocardial cell fates were examined in embryos treated with DMSO (A1-C1), or with SB-431542 at various time points (A2-C5). (A2-C2) Embryos treated at 4 h, express small amounts of *amhc *and *cmlc2*, but not *vmhc *(arrows). (A3-3) *cmlc2*, *amhc *and *vmhc *are bilaterally expressed in embryos treated at 4.3 h. (A4-C5) All heart markers are expressed at the midline in embryos treated at 4.7 h. Images are dorsal views at 24 h, anterior to the left.

We next asked if Nodal signals act similarly in the ventrolateral margin. The teleost heart is composed of two chambers, the atrium and ventricle, which express *atrial myosin heavy chain *(*amhc*) and *ventricular myosin heavy chain *(*vmhc*), respectively (Fig. 7B1, C1) [49]. Both chambers express *cardiac myosin light chain 2 *(*cmlc2*) (Fig. 7A1). Despite a large region of overlap, atrial myocardial precursors can be found in more animal locations and ventricular myocardial precursors are located closer to the margin. We found that *cmlc2 *and *amhc *expression are first detected when Nodal signaling is blocked at 4 h (Fig. 7A2, B2 arrows; N = 16/20 and N = 18/21, respectively). *vmhc *expression is never observed at this time point, indicating that specification of atrial myocardium precedes specification of ventricular myocardium (Fig. 7C2; N = 0/19). *vmhc *expression is first observed in embryos treated at 4.3 h (Fig. 7C3, N = 24/27). The short delay between specification of atrial myocardial precursors and ventricular myocardium is consistent with the small number of atrial progenitors located in animal cells where ventricle myocardial is not found [7]. The bilateral expression of heart myocardial genes in embryos treated at 4.3 h is consistent with the lack of endoderm at this stage (Fig. 7A3-C3) [50]. Myocardial precursors fuse into a tube at the midline when Nodal signals are blocked at 4.7 h, when we first observe beating hearts in live embryos (Fig. 7A4-C4; Fig. 5). We conclude that within the presumptive mesoderm and endoderm, marginal cell types require longer periods of Nodal signaling than other cell types.

### Nodal levels control when cell fates are specified

**Figure 8:**
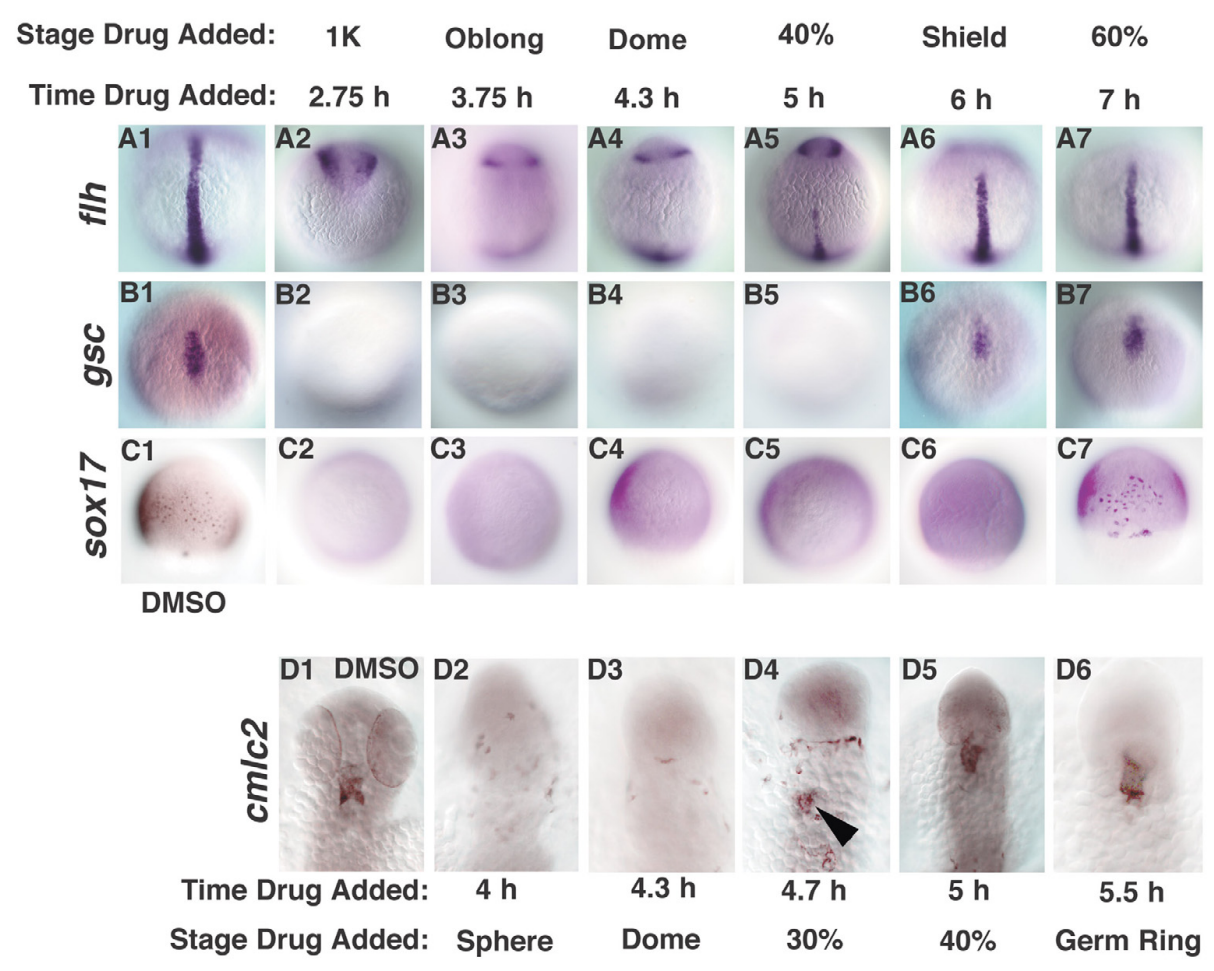
**Cell fate specification is delayed *squint *mutants**. Cell fates were examined in *sqt *mutant embryos treated with DMSO (A1-D1), or with SB-431542 at various time points. (A1-7) *flh *was first expressed at the midline in embryos treated at 5 h (A5). (B2-7) *gsc *expression is first detected in embryos treated at 6 h (B6). (C2-7) *sox17 *expression is first detected when embryos are treated at 7 h (C7). (D1-7) *cmlc2 *expression was first detected in embryos treated 4.7 h (D4, arrowhead). Dorsal views of 10 h (A1-B7), 8 h (C1-C7) or 24 h (D1-D6). In D1-D6, anterior is up. The embryos in Figs. 8 and 9 are from the same clutch and weretreated in parallel, along with wild type controls (not shown).

We have shown that Nodal signals specify different cell types within the mesoderm and endoderm at different times, although there is a period during which they specify multiple tissues simultaneously. This could be explained if the responding cells have fixed time windows during which they need to be exposed to Nodal signals in order to adopt particular fates. If so, then mesoderm and endodermal cell fates will be specified at the same time as wild type even when the Nodal dose is reduced. To test this, we asked when cell fates are specified in *sqt *mutants, which have reduced levels of Nodal signaling. We found that *flh *expression at the midline was only observed when Nodal signaling was blocked at 5 h in *sqt *mutants (Fig. 8A5; N = 15), as opposed to 4 hr in wild type (Fig. 4B6). Thus, notochord specification is delayed by an hour in *sqt *mutants. Specification of the prechordal plate and endoderm are also delayed in *sqt *mutants. *gsc *expression is only apparent in *sqt *mutants treated at the onset of gastrulation (6 h) (Fig. 8B6, N = 21; compare with Fig. 6C4), and *sox17 *expression is first apparent in embryos treated at 7 h (Fig. 8C7, N = 16/20; compare with Fig. 6E5). We also observed a delay in specification of ventrolateral cell types in *sqt *mutants, since *cmlc2 *expression is only apparent in embryos treated at 4.7 h (Fig. 8D4, arrowhead, N = 12/16; compare with Fig. 7A2). These results rule out the possibility that presumptive mesoderm and endodermal cells have discrete windows of competence that determine their response to Nodal signals.

**Figure 9:**
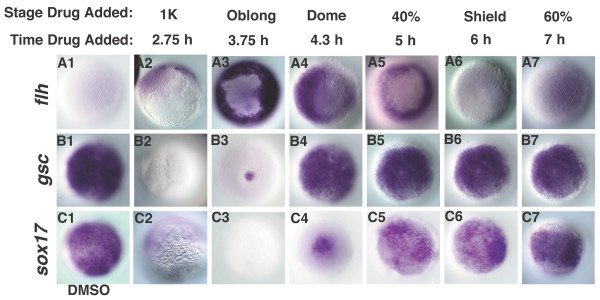
**Cell fate specification is accelerated when Nodal levels are increased**. Embryos were injected with *sqt *mRNA at the 1–4 cell stage and treated with DMSO (A1-C1) or SB-431542 at various time points (A2-C7). Sqt induces ubiquitous expression of *gsc *and *sox17 *(B1, C1) but not *flh *(A1). (A2-C2) SB-431542 treatment at MBT blocks expression of each of these markers. *flh *is strongly expressed in embryos treated at 3.7 h (A3), but fades at later time points (A4-7). *gsc *expression is first detected in embryos treated at 3.7 h (B3), and expands at later time points (B4-7).* sox17 *expressing cells are first detected when embryos are treated at 4.3 h (C4), and expands at later time points (C5-7). Animal pole views at 10 h.

The delay in cell fate specification in *sqt *mutants suggests that Nodal levels control when cells fates are specified. If so, then specification of mesodermal and endodermal cell types should be accelerated when Nodal levels are increased. To test this, we examined *flh*, *gsc *and *sox17 *expression in embryos injected with *sqt *mRNA and treated with SB-431542 at different time points after MBT. *flh *expression was not detected in control embryos (Fig. 9A1), but *gsc *and *sox17 *were both expressed ubiquitously (Fig. 9B1, C1). Expression of all three genes was inhibited when we blocked Nodal receptor activity at MBT (Fig. 9A2-C2). *flh *was broadly expressed in embryos treated at 3.7 h (N = 14), but gaps are often apparent at the animal pole. This indicates that the notochord is specified earlier in embryos with elevated Nodal signals than in wild type (Fig. 9A3, compare with Fig. 4B6). Similarly, specification of both prechordal plate and endoderm occur earlier in embryos with elevated Sqt. *gsc *is first detected in embryos treated at 3.7 h, as opposed to 4.3 h in wild type (Fig. 9B3, N = 10/19; compare with Fig. 6C4), and is ubiquitously expressed in all embryos treated at 4.3 h (Fig. 9B4; N = 12). This indicates that specification of prechordal plate is greatly accelerated when Nodal signaling is elevated. *sox17 *is first observed in embryos treated at 4.3 h instead of 5 h in wild type, representing a slight acceleration in endoderm specification as compared to wild type (Fig. 9C4, N = 10/12; Fig. 6E5). These results show that the level of Nodal signaling determines when mesoderm and endodermal cell fates are specified.

According to the "ratchet-model", cells generate a response appropriate to the highest dose to which they are exposed independently of the duration of exposure [33]. If true, then cells should always adopt the most marginal fate when they are exposed to a uniformly high Nodal dose, regardless of how long the exposure lasts. In contrast to this prediction, however, we found that cells in Sqt-injected embryos are transiently specified to the more animal *flh *expressing fate (Fig. 9A4). As the duration of exposure increases, *flh *expression gradually diminishes (Fig. 9A4-A7), and *gsc *and *sox17 *expression increase concomitantly (Fig. 9B4-C7). This demonstrates that cells adopt progressively more marginal identities in response to increasing exposure times to Nodal signals. These results rule out the possibility that presumptive mesoderm and endodermal cells respond to Nodal signals by a ratcheting-type mechanism.

## Discussion

### Chemical inhibitors of ALK 4/5/7 are new tools for dissecting the roles of Nodal signals

In this study, we addressed the question of when members of the Nodal-related subclass of the TGF-β superfamily act to pattern the mesoderm and endoderm. We took a pharmacological approach to inactivate Nodal signaling at different times, and examined the resulting cell fates by an extensive analysis of gene expression and morphology. Three lines of evidence show that we were able to inhibit zygotically expressed Nodal signals. Firstly, we generated a phenocopy of *sqt; cyc *double mutants by treating embryos with 800 μM SB-431542 at the mid-blastula stage, when zygotic expression of *sqt *and *cyc *initiates (Fig. 1). Secondly, we could phenocopy *cyc *single mutants by treating embryos at the onset of gastrulation, when *cyc *expression predominates (Fig. 4). These two experiments demonstrate that our treatment reduces receptor activity to at least the levels in the respective mutants. We confirmed our results with a second drug, SB-505124, which is more potent and soluble than SB-431542 (Fig. 1; data not shown), which rules out possible artefacts due to the high dose of SB-431542. Finally, drug treatment in the late blastula stages inhibited expression of a Nodal target gene within 30 minutes (Fig. 2).

Our results differ markedly from those of earlier studies, in which 50 μM SB-431542 was unable to reproduce the *sqt; cyc *phenotype when added to embryos older than the 8-cell stage [40,41]. Two technical aspects of our treatment protocol may account for our different results. First, we used a much higher dose of SB-431542 (800 μM) than the other groups. Secondly, we perforated the embryos to ensure the drug fully penetrated the embryos. Perforation was not necessary with SB-505124, which was also effective at a much lower dose (50 μM). We conclude that the milder effects of the drug reported by others are due to the poor ability of SB-431542 to penetrate the embryo as the number of cells increases during the cleavage stages. Even though multiple ligands can activate the ALK4/5 and 7 receptors, our phenotypes all resemble those resulting from reductions of *nodal-related *gene function [17,23]. This indicates that the other Activin-like ligands are either not expressed during the stages we examined or act downstream of Nodal signals.

### Time-dependent patterning of the animal-vegetal axis by Nodal signals

Previous attempts to determine when Nodal signals specify different mesoderm and endoderm cell types have focused on the analysis of *oep *mutant embryos. In *Zoep *mutants, late Nodal signaling is blocked due to the absence of an essential co-receptor, and prechordal plate and endoderm do not form [32,51]. It is not clear, however, whether these defects are due to the absence of late Nodal-signaling activity, or to the reduction of signaling levels caused by the decay of maternally supplied Oep protein. In an alternate approach to determine the role of Nodal signals at different times, *oep *function was restored to *MZoep *mutants at different stages, rescuing the ability of mutant cells to respond to Nodal signals [31,52]. In these experiments, restoring Nodal signaling at early stages completely rescued *MZoep *mutants. By contrast, prechordal plate and endoderm was missing when Nodal signaling was restored at later stages. Although these results are apparently consistent with our findings, we found that *sqt *and *cyc *expression are expressed at very low levels when *oep *function is supplied at late stages (4 h; Hagos and Dougan, submitted). Since the defects in late-rescued *MZoep *mutants result from aberrant *nodal-related *gene expression, these experiments do not address the question of when Nodal signals are required to specify cell fates.

By conditionally inactivating the Nodal receptors, we were able to determine the specification state of the presumptive mesoderm and endoderm at different embryonic stages. We found a time-dependent progression of cell fate specification along the animal-vegetal axis, consistent with earlier studies demonstrating that Nodal signals pattern the animal-vegetal axis, but not the dorsoventral axis [17]. Blocking Nodal signals at late stages inhibits formation of tissues derived from the margin, such as prechordal plate and endoderm, but not from more animal regions, such as notochord or somites (Figs. 4, 5, 6). Previous studies have determined that endoderm and prechordal plate require higher doses of Nodal signals than somites [17,31]. This suggests a linkage between Nodal dosage and the length of exposure.

### Nodal levels control when cells are specified to become mesoderm and endoderm

Our results place Nodal signals at the top of a developmental program that determines the fates of responding cells and controls when these fates are specified. We considered the possibility that Nodal signals pattern the mesoderm and endoderm by acting in fixed time windows to specify different cell types. When Nodal levels are low, as in *sqt *mutants, specification of endoderm does not begin until early gastrulation (7 h; Fig. 8C7). By contrast, when Nodal levels are high, specification of endoderm begins 1.7 h earlier (Fig. 9C4). We conclude that cell identities are specified at different times depending on the Nodal dosage (Figs. 8, 9). These results exclude the possibility that cells have fixed time windows during which they can adopt particular mesoderm and endodermal fates in response to Nodal signals. To the contrary, the level of Nodal signalling determines when cells are specified to adopt particular mesoderm and endodermal identities.

Previous cell transplant experiments defined a broad window of competence during which cells can respond to mesoderm and endoderm inducing signals, which we now know to be the Nodal-related proteins [53,54]. Experiments in *Xenopus *animal caps demonstrated that this window of competence is controlled by an intrinsic timing mechanism and ends by mid-gastrulation [55]. Our results show that within this broad window, cells have a considerable degree of flexibility as to when they can become mesoderm and endoderm that depends on the levels of Nodal signals. At the molecular level, the loss of the ability to respond to Nodal signals could reflect the Nodal-dependent induction of a feedback inhibitor of the pathway. Consistent with this idea, expression of the secreted Nodal antagonist Lefty is under the control of Nodal signaling [23,56]. Thus, one role of Lefty could be to place a temporal limit on when cells can respond to Nodal signals. In support of this, Nodal signals persist well into gastrulation when *lefty *function is depleted, and act during this time to convert ectoderm into mesoderm and endoderm (X. Fan and S. Dougan, unpublished data) [26,57].

### The length of exposure to Nodal signals determines cell fate choices

**Figure 10:**
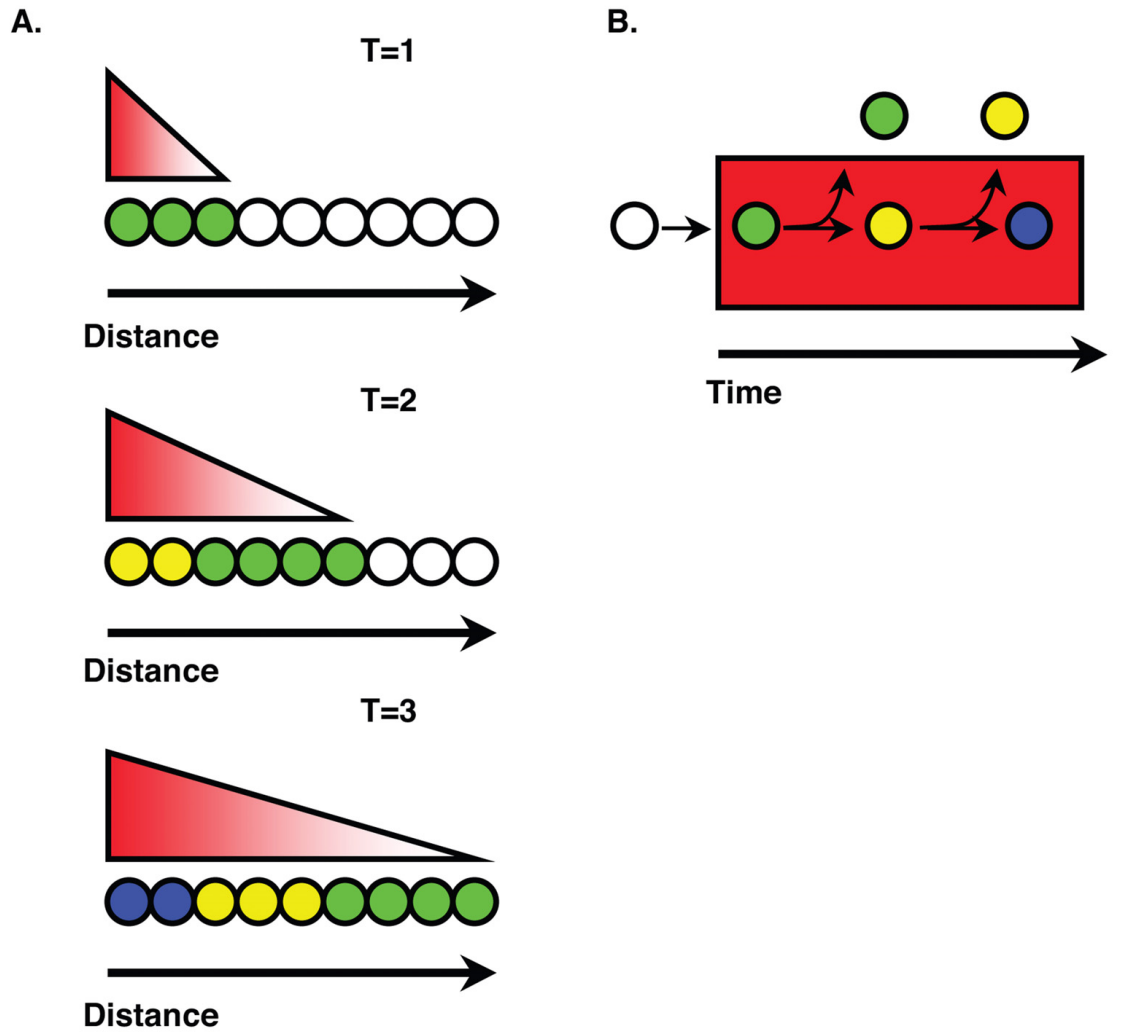
**Models for forming a gradient of Nodal signals over time**. In the ratchet model (A), the morphogen spreads through a tissue over time and cells receive different Nodal doses at different times. Cells respond to the highest dose to which they are exposed. In the spatio-temporal gradient model (B), cells freely move into (not depicted) and out of (arrows) a zone of signaling activity (red rectangle). A short residence time in this zone determines a low cumulative dose of Nodal signals (green circle), whereas longer residence times determine higher cumulative Nodal dose (yellow and blue circles).

Cells are exquisitely sensitive to the dose of Activin-like signals. Experiments with dissociated *Xenopus *animal cap cells showed that as few as 100 molecules of Activin induce expression of the pan-mesodermal marker, *Xbra*, whereas 300 bound molecules induce *gsc *expression [35]. In these experiments, cells were exposed to different doses of Activin for 10 minutes and changes in cell fate were assessed hours later. The results supported the view that cells "ratchet-up" their response as a morphogen gradient is established and the dose of Activin crosses particular threshold levels (Fig. 10A)[33]. Because the length of exposure was constant, these experiments did not address the effects of prolonged exposure to Activin on cell fate decisions. In our experiments, by contrast, we examined the response to the endogenous mesoderm and endoderm inducing signals in whole zebrafish embryos. Marginal cells were continuously exposed to Nodal signals until we blocked the response by drug treatment. Our results emphasize the importance of the length of exposure in determining the overall dose and consequent fate choice. Importantly, all mesoderm and endodermal cell types are present in *sqt *mutants, but are specified at later times than in wild type. The only Nodal-related protein in these embryos is Cyc, which has a shorter range than Sqt and is expressed at reduced levels in *sqt *mutants [17,25]. Thus, prolonged exposure to low doses of a signal compensates for the overall reduction of levels. Furthermore, the long-range action of a secreted factor is not essential for normal development of the two germ layers in zebrafish.

### Generating a response to a cumulative Nodal dose

We found that cells respond to the cumulative dose of Nodal signals to which they are exposed. In embryos exposed to a uniform, high Nodal dose, cells exhibit a time dependent transformation towards more marginal fates as the length of exposure increases (Fig. 9). This means that cells must have a mechanism to record the duration of their exposure to Nodal signaling and to generate a response to the cumulative dose. Although this regulation may occur at many different levels, the ultimate readout is at the level of gene transcription. Of the marker genes we analyzed, *gsc *is a likely direct target of the Nodal pathway [58]. *gsc *expression initiates at 4 h in the absence of both *sqt *and *cyc *function, but quickly decreases [17]. This indicates that Nodal signals are required for maintenance, but not for the induction of *gsc *expression. In this study, we showed that *gsc *expression is lost when Nodal signaling is inactivated at 4.3 h, but continues when Nodal signaling is blocked at 5 h (Fig. 6). Thus, Nodal input is required for about an hour in order to maintain *gsc *expression. After this transient maintenance phase, *gsc *expression continues independently of Nodal, by an unknown mechanism. In *sqt *mutants, it takes a longer period of time for the *gsc *promoter to transit to the Nodal independent phase, whereas the *gsc *promoter reaches this state more rapidly when Sqt is overexpressed. Other genes have been shown to undergo similar phases of gene regulation, most notably the *Drosophila engrailed *gene [59], but this is the first case to our knowledge in which the levels of a secreted factor control the length of the maintenance phase of a target gene.

### A spatio-temporal gradient model for patterning by Nodal signals

Any model for how Nodal signals act to pattern the mesoderm and endoderm must account for four observations. First, the model must explain how adjacent cells become exposed to different levels of Nodal signals. Fate mapping studies show that precursors of cell types that require different levels of Nodal signaling, such as somites and endoderm, are juxtaposed in the pre-gastrula stage embryo [5,17]. Second, the model must account for our observation that the blastomeres are highly dynamic during the period they respond to Nodal signals. We found that Nodal signals act primarily before 5 h (40% epiboly) (Figs. 4, 5, 6), a period in which cells divide rapidly and frequently change positions with respect to each other [60]. This presents a particular challenge to classic morphogen gradient models, which generally assume a static field of responding cells. Third, the model must explain how a short-range signal, like Cyc, can specify the same cell types as a long-range signal, like Sqt. Finally, the model must account for our observation that cells respond to the cumulative dose of Nodal signals.

We propose that the total Nodal dose is a function of both the length of time a cell is exposed to Nodal signals and the distance of a cell from the Nodal source (Fig. 10B). Key predictions of this model remain to be tested, but it accounts for all these observations. In this view, cells that remain near the Nodal source for an extended period would receive a high dose and adopt a marginal cell fate, such as prechordal plate or definitive endoderm. Conversely, cells that move away from the source after a short time would receive a lower dose and become somites. Specification of mesoderm and endoderm is delayed in *sqt *mutants because it takes longer than in wild type for cells to accumulate the necessary Nodal dosage. Because the gradient of positional information is influenced by the length of time responding cells are exposed to the signaling source and their distance from the source, we call this the spatio-temporal gradient model. In other species, Nodal signals also pattern tissues comprised of dynamic cell populations, such as the node and primitive streak in mice and Hensen's node in the chicken [61,62]. Thus, cell movements could provide a general mechanism for generating a gradient of exposure to Nodal signals during mesoderm patterning in all vertebrates.

Our model predicts that a stable source of Nodal signals exists in the embryo that is independent of the dynamic cell movements of the responding cell population. We propose that the extraembryonic yolk syncytial layer (YSL) acts as this source. Sqt is normally expressed in this tissue and can induce fate changes in overlying blastomeres when overexpressed in the YSL [19,24]. We suggest that Nodal signals in the YSL act to induce and/or maintain *nodal-related *gene expression in the overlying blastomeres via the autoregulatory pathway. If a cell that is initially close to the YSL moves away, it will lose expression of *sqt *and *cyc*. Conversely, *sqt *and *cyc *expression will be induced in a cell as it moves closer to the YSL. Thus, the autoregulatory pathway provides a mechanism by which a stable zone of Nodal signaling can be imposed upon the dynamic, intermixing population of cells at the embryo margin.

## Conclusion

Our data indicate that Nodal signals act in a time-dependent manner to pattern the mesoderm and endoderm. Three lines of evidence support the idea that cells respond to the cumulative dose of Nodal signals. First, marginal cell types, which are specified by the highest Nodal dose, require the longest exposure to Nodal signals. Second, cell fate specification is delayed when Nodal levels are reduced, and accelerated when Nodal levels are increased. Finally, in response to a uniform, high Nodal dose, cell fates transform toward progressively more marginal identities as the length of exposure increases. These results rule out the possibility that Nodal signals act during discrete time windows to specify different mesodermal and endodermal cell types. They are also inconsistent with the "ratcheting-up" model, in which the absolute number of occupied receptors determines cell fates, not the duration of exposure. We conclude that cells respond to the cumulative Nodal dose, which we suggest is a product of the distance of the responding cell from the signaling center and the length of exposure.

## Methods

### Zebrafish strains and staging

We used the WIK strain to obtain wild type embryos. Embryos homozygous for the *sqt*^*cz35 *^null allele were obtained from crossing mutant adults. *oep*^*tz57 *^mutant adults were obtained by mRNA injection, as previously described [10]. In all experiments, the embryonic stages were determined by morphology and are reported as hours postfertilization (h) at 28.5°C, according to Kimmel et al. (1995).

### Drug treatment

SB-431542 (4- [4-(1,3-benzodioxol-5-yl)-5-(2-pyridinyl-1*H*-imidazol-2- yl]benzamide), was obtained from Tocris (Ellisville, MO) and stored as a 100mM stock in DMSO at -20°C. SB-505124 (2-(5-benzo[1,3]dioxol-5-yl-2-tert-butyl-3Himidazol- 4-yl)-6-methylpyridine hydrochloride) was a kind gift from GlaxoSmithKline (King of Prussia, PA) and is stored at 10 mM in DMSO at 4°C. For the drug time course studies shown in Figs. 1 and 4, approximately 1000 embryos equivalently staged embryos from 3–4 single pair matings were pooled, split into 10 dishes at a density of 100 embryos/dish, and raised in an incubator at 28.5°C. For drug treatment, embryos from one dish were removed at the desired stage, perforated near the margin with a pulled capillary tube, and split into glass dishes containing the drug in 5 ml embryo medium, at a density of 25 embryos/dish. Embryos were fixed at 10h and split into three groups for analysis of *ntl*, *flh *or *shhb *expression, or fixed at 14h and split into two groups for analysis of *MyoD *or *pax2.1*. Time courses depicted in other figures followed the same protocol, but embryos were fixed at the stages indicated for analysis of marker gene expression. In each figure, representative images are shown, and all embryos were treated on the same day. Embryos damaged by the perforation were discarded. Embryos treated with SB-505124 did not require perforation. In all experiments, some embryos in each experiment were allowed to develop until 24 h and examined morphologically to verify the efficacy of the treatment. All experiments were performed at least two times. The effective dose on 2.75 h embryos SB-431542 was determined in a titration of 5 μM-1mM SB-431542 or 3 μM–75 μM SB-505124. SB-431542 treatment was always associated with the formation of a dark precipitate in the solution. At 800 μM, all embryos resembled *sqt; cyc *mutants, whereas lower doses generated milder phenotypes similar to *Zoep *mutants [51]. This milder phenotype is also observed by treating cleavage stage embryos with 50 μM SB-431542 (data not shown) [40,41]. The previously described toxic effects of SB-431542 in cell culture are apparent at doses above 800 μM on blastula stage embryos and above 100 μM on cleavage stage embryos (data not shown) [38]. For SB-505124, the lowest dose that produced the *sqt; cyc *phenotype ranged from 30–50 μM, depending on the age of the drug.

### Microinjections and whole-mount *in situ *hybridization

The *sOep, sqt *and *TARAM-D *cDNAs were described previously [19,32,45]. Sense transcripts were synthesized using the Message Machine kit (Ambion, Inc., Austin TX). We injected 10pg *sqt*, *TARAM-D *or *β-galactosidase *mRNA into chorionated embryos at the 1–4 cell stage. 100pg *sOep *mRNA was co-injected into the YSL of *MZoep *mutants with the Oregon Green 488 lineage tracer dye (Invitrogen, Inc., Carlsbad, CA) to verify the targeting of the injection, as described [31]. *In situ *hybridizations were performed as in Dougan, et al., 2003. We used the following probes: *sqt*, *cyc*, *gsc*, *ntl*, *flh*, *MyoD*, *pax2.1*, *shhb*, *sox17*, *mezzo*, *cyp26*, *cmlc2*, *amhc *and *vmhc *[16,19,23,46-49,63-69].

## Authors' contributions

EH carried out the molecular genetic studies and SD conceived of the study and participated in its design. Both authors jointly drafted the manuscript. The final manuscript was approved by both authors.
